# Publishing Protocols for Partnered Research

**DOI:** 10.1007/s11606-014-3037-0

**Published:** 2014-10-30

**Authors:** Sylvia J. Hysong, LeChauncy Woodard, Jennifer H. Garvin, Jeffrey Murawsky, Laura A. Petersen

**Affiliations:** 1 Houston VA HSR&D Center for Innovations in Quality, Effectiveness and Safety, Michael E. DeBakey VA Medical Center, Houston, TX USA; 2 Baylor College of Medicine, Houston, TX USA; 3 Salt Lake City Veterans Health Care System, Salt Lake City, UT USA; 4 University of Utah, Salt Lake City, UT USA; 5 Veterans Integrated Service Network Great Lakes (12), Westchester, IL USA; 6 Loyola University Stritch School of Medicine, Maywood, IL USA

**Keywords:** Partnership research, Reporting standards, Research protocols

## Abstract

Published scientific protocols are advocated as a means of controlling bias in research reporting. Indeed, many journals require a study protocol with manuscript submission. However, publishing protocols of *partnered* research (PPR) can be challenging in light of the research model’s dynamic nature, especially as no current reporting standards exist. Nevertheless, as these protocols become more prevalent, a priori documentation of methods in partnered research studies becomes increasingly important. Using as illustration a suite of studies aimed at improving coordination and communication in the primary care setting, we sought to identify challenges in publishing PPR relative to traditional designs, present alternative solutions to PPR publication, and propose an initial checklist of content to be included in protocols of partnered research. Challenges to publishing PPR include reporting details of research components intended to be co-created with operational partners, changes to sampling and entry strategy, and alignment of scientific and operational goals. Proposed solutions include emulating reporting standards of qualitative research, participatory action research, and adaptive trial designs, as well as embracing technological tools that facilitate publishing adaptive protocols, with version histories that are able to be updated as major protocol changes occur. Finally, we present a proposed checklist of reporting elements for partnered research protocols.

Published standardized scientific protocols are advocated for controlling bias in research reporting. Evidence from randomized controlled trials (RCTs) is often synthesized for various purposes, including formulation of clinical practice guidelines and justification of payment by insurers, and therefore, transparency of methods in these trials is essential. The strength of evidence is often determined by the quality of methods, which in turn reflects upon the generalizability of findings.^1^^,^ 2 Indeed, journals often require a study protocol in conjunction with manuscript submission to substantiate evidence presented in research findings.^3^^,^ 4

Increasingly, health care research is turning to partnered research models to expedite translation of research into practice and to elicit the practice-based evidence often missing from traditional RCTs. As these methods become more prevalent, publishing a priori protocols of partnered research studies becomes increasingly important. However, due to the dynamic nature of this research model, publishing a research protocol can be challenging, especially as no current reporting standards exist. Using as illustration a suite of studies aimed at improving primary care coordination and communication, we identify challenges in publishing protocols of partnered research relative to traditional designs, present potential solutions to said challenges, and propose an initial checklist of content for inclusion in protocols of partnered research.

## CHALLENGES IN PUBLISHING PARTNERED RESEARCH STUDY PROTOCOLS

Publishing protocols of partnered research is consistent with goals advocated by the CONSORT statement, the nationally accepted standard for reporting traditional RCT findings.^1^ However, because of the dynamic nature of partnered research as well as the differences between scientific and operational goals of research, many protocol elements recommended in the CONSORT statement are often difficult to report. We illustrate two of these challenges and potential solutions in a suite of studies currently underway, led by the Houston Center for Innovations in Quality, Effectiveness and Safety. The objective of the studies is the implementation of interventions to enhance communication and coordination of primary care in Patient-Aligned Care Team (PACT) settings, the Veterans Administration’s (VA’s) model of the patient-centered medical home. To accomplish this, we partnered with the Great Lakes VA Healthcare System (known within the VA as Veterans Integrated Service Network (VISN) 12, or Network 12) and four other clinical and operational partners (see acknowledgments).

### Challenge #1: Partnered Research is Highly Dynamic

Partly because the intent of partnered research is to accelerate and optimize findings to meet operational and clinical needs, the approach and methodology often evolve as the study progresses. This can pose significant reporting challenges for study protocols. In this first case example, we briefly describe key details of one of our studies, which is focused on developing care coordination measures to identify point-of-care information needs for improved coordination, and describe proposed solutions to this challenge.

#### Study A: Coordination Measure Development

PACTs have been proposed as one of multiple strategies to improve care coordination. For said strategy to succeed, however, team members must be effective in the act of coordinating: working collectively on interdependent tasks to deliver evidence-based care that could not be accomplished as effectively by a single provider. To arrive at a consensus of what constitutes good care coordination, this study will develop coordination measures collaboratively and iteratively with frontline PACT members from Network 12, including providers, nursing staff, extended PACT members, and other key stakeholders such as information technology staff. We will then conduct focus groups comprising PACT members and key stakeholders, who will help identify what is needed at the point of care in order to coordinate successfully to the standards detailed by the measures. Ultimately, we will develop feedback reports of participants’ performance on the newly developed measures (also in collaboration with Network 12) to explore the change in care coordination quality.

Study A (coordination measure development) uses a systematized process to develop measures of care coordination; however, the measures have not yet been developed, despite representing a primary outcome of the study. Similarly, the main intervention of the study, the coordination feedback report, will be created collaboratively with Network 12 and tailored to meet their needs. These details would normally be reported a priori in a traditional research protocol, yet remain unspecified in a partnered research protocol.

Additionally, although site-selection criteria were initially included in the study, site selection was ultimately coordinated with Network 12 and the other studies in this suite of projects so as to avoid adversely impacting any given facility. One selected site is a remote outpatient clinic, differing significantly in size, configuration, and resources from both its parent medical center and more urban outpatient clinics. Though this may impact the internal and external validity of the findings, from an operational standpoint, this clinic was ideally suited for the investment the time and resources required for the study.

### Challenge #2: Scientific and Operational Goals Are Not Always Aligned

Traditional scientific research has as its central goal the discovery of generalizable knowledge, with careful control and replicability as central tools, albeit sometimes at the expense of expediency. Conversely, operational partners engage in research activities to answer specific questions to help them immediately improve their practices, albeit sometimes at the expense of replicability and generalizability. Such misalignment of goals has considerable implications for sampling, data quality, and the ability to report these details in a protocol. The next two studies—one focusing on point-of-care health literacy and activation information to improve diabetes care (Study B), and the other with the purpose of automating heart failure data for treatment goals at the point of care (Study C)—illustrate several of these challenges.

#### Study B: Diabetes Goal Setting

The purpose of this study is to implement an evidence-based diabetes goal-setting intervention, tailored to individual levels of health literacy and activation, in patients with treated but uncontrolled diabetes receiving care within Network 12 PACTs. Although the importance of self-management by patients with diabetes is widely accepted, providing patients with effective self-management training and support can be challenging due to the time constraints in primary care encounters and limited clinician training in behavior modification. Previously, we demonstrated the effectiveness of an intervention (Empowering Patients in Chronic Care) to help patients set highly effective evidence-based goals on both diabetes self-efficacy and hemoglobin (Hb) A1c levels.^5^ The current study evaluates the process of implementing this collaborative goal-setting intervention, personalized to patient activation and health literacy levels, into routine Network 12 PACT care and to evaluate its effectiveness relative to usual care.

Study B focuses on patients who, despite being engaged in care, have poorly controlled diabetes. One primary outcome is glycemic control, traditionally defined using guideline-recommended thresholds. However, our partner network has actively engaged in shared decision-making around HbA1c goals. This approach to establishing glycemic-control goals is currently a high priority for the network, with HbA1c goals determined based on individual patient characteristics rather than solely dictated by national guidelines. We are working with VISN 12 to adapt our methods to reflect these negotiated goals, and thus we could not include a detailed description of this approach in a research protocol a priori.

#### Study C: A Heart Failure Communication Aid

This study’s primary goal is to provide an accurate, effective communication aid to improve beta-blocker titration consistent with guideline-recommended care. The study uses natural language processing (NLP) to extract relevant chart data for beta-blocker titration in patients, post-hospital discharge, for chronic heart failure exacerbation. The study team then summarizes and provides PACT team members key information regarding beta-blocker titration. Guided by the Promoting Action on Research Implementation in Health Services (PARIHS) model,^6^^,^ 7 the study team will identify the most appropriate data elements to include and determine the best way to present them to PACT providers. The communication aid will then be tailored by employing human factors principles and co-creating its design with our stakeholders (informed visual design). Finally, the team will evaluate the aid’s effectiveness at improving guideline-recommended beta-blocker titration, where clinically appropriate, and at improving clinical outcomes in patients with chronic heart failure.

The NLP methods used in Study C require a power analysis to determine the number of documents needed for training the system to recognize and parse text correctly. This usually requires a specific number of patients from a given number of participating medical facilities. In partnered research, where facilities have greater latitude regarding whether to participate and to what degree, scientific and operational needs can be misaligned such that facilities may need to limit participation because of perceived burdens on staff. Therefore, although the document requirements are scientifically determined through power analysis, limited site participation may mean having fewer than the required number of documents. To address this misalignment, we use the available number of documents, then determine the actual power post hoc, and report it as a limitation if necessary.

## OVERCOMING CHALLENGES TO PUBLISHING PROTOCOLS OF PARTNERED RESEARCH

The challenges discussed in this article are not trivial; fortunately, we can draw upon other disciplines, such as qualitative methods, participatory action research, and implementation science, to devise solutions for these challenges.

### Borrow a Page from Qualitative and Participatory Action Research

Because of its iterative nature during data collection and analysis, rigorous qualitative research employs numerous techniques for ensuring transparency, including documentation of sampling strategies and analytic processes (e.g., development of coding strategies, analyst training, etc.), stopping rules for reaching sample saturation, and procedures for ensuring study quality (e.g., negative case analysis, use of multiple perspectives in both data collection and analysis). For example, Smith and colleagues^8^ recommend focusing on “who”, “what”, where, “when”, and “how” in reporting the project design and process of a participatory action research project. Study A (Coordination Measure Development), which follows explicit procedures for measure development, can benefit from this approach. Additionally, examples of possible resulting measures could be reported to allow for greater transparency with regard to their potential impact on other study components. Indeed, Leykum and colleagues suggest integrating such design elements into more traditional research design—for example, a randomized controlled trial could improve intervention implementation success.^9^

### Look to Adaptive Trial Designs for Reporting Guidance

According to the Patient Centered Outcomes Research Institute (PCORI), “An adaptive trial is one in which key trial characteristics…evolve according to prespecified rules during the trial, in response to information accruing within the trial itself.”^10^ PCORI recommends that adaptive trials follow CONSORT guidelines, with some modifications. These include rules for stopping for futility or early success, procedures for sample size re-estimation, and documentation of procedures for transitioning between each stage of the study. Adaptive trial reporting conventions have already begun to see use in comparative effectiveness studies of classic health services research interventions such as hypertension management,^11^ a first step towards more widespread adoption of this type of reporting, such as in partnered research.

Approaches such as these can be particularly beneficial for Study C (Heart Failure Communication Aid), in which sample size calculations for testing the information extraction system rely on the prevalence of data elements (i.e., targeted concepts): if the prevalence of a concept is low, training the system generally requires more documents. Thus, if the number of documents differs sufficiently from initial projections, it may be necessary to re-estimate sample size. Alternatively, if the prevalence of data elements is higher than anticipated, training may require fewer documents, and therefore may have to stop earlier than anticipated due to reaching a prespecified level of accuracy (e.g., 95%). Stopping rules such as these can be specified in the protocol.

### Embrace Technology

Current advances in electronic formats of journals could address some of these challenges of partnered research. Amendable protocols with version histories could be updated as major protocol changes occur. A disadvantage would be the burden on journals or protocol repositories of peer-reviewing each update. An alternative approach could involve providing links to a study website where such version histories and changes could be documented. Such a strategy could address Study B’s (Diabetes Goal Setting) ongoing communication and feedback needs from multidisciplinary PACT clinicians and study participants. The study website being developed for this project will include clinician and patient manuals, training materials, and importantly, study updates. Modifications to study procedures derived from partner feedback will be updated on the study website, which will be used by clinicians leading the coaching sessions.

## SUGGESTED REPORTING FOR PARTNERED RESEARCH PROTOCOLS

Table 1 presents an initial set of elements that we propose for inclusion in a partnered research protocol. This list is not exhaustive; rather, it is a starting point for developing a comprehensive approach to reporting partnered research. Such a protocol can be useful in several ways, including addressing some of the reporting challenges discussed in this article, approaching partners about the research, and working with partners during the conduct of the study. For example, reporting a development plan for an undeveloped intervention (elements 6c–d in Table 1) can help address the challenge of reporting dynamic components of the research. In addition, research usually contains parts that are immutable and parts that can be modified over time; for example, a design calling for randomization to two different treatment approaches is usually not mutable, though the treatment itself might be adapted locally to some extent. Explicitly documenting and explaining this dichotomy to partners is often helpful for optimally aligning research and operational goals.

**Table 1:** Proposed Checklist for Partnered Research Protocols

Element	Description
1. Background	
a. Problem statement	
b. Conceptual framework	
c. Objectives of study/project	Include both scientific objectives and operational objectives, if these are different.
2. Project description and study design	What is the basic design for evaluating the project or answering the research question of interest (consult EQUATOR for guidance in reporting specific types of study designs)? Provide also a brief description of the impetus for the project, as well as overall time frame.
3. Key personnel and partnership approach	
**a. Rationale for partnership approach**	**Why is the research question of interest best or uniquely answered through a partnered research design**?
*b. Research team*	*What is the professional background of each member of the research team*? *What relevant experience and*/*or training does he*/*she bring to the study*?
**c. Operational partners**	**Who are the operational partners in the study**? **What is their role in the study**? **How does the research benefit the operational partners**?
**d. Relationship between research team and operational partners**	**e.g**., **How long have they been working together**? **Is one subordinate to the other**, **or are they independent**? **How will the research team engage key stakeholders from the operational partner**?
*e. Management strategy between partners and research team*	*How will the research team and the partners maintain communications*, *solve problems*, *and resolve conflicts throughout the life of the project?*
4. Study setting	Eligibility criteria for site/setting selection or desired site characteristics; **operational partner role in selection process**; discussion of scientific criteria vs. operational criteria, and strategy for reconciliation if these differ; if setting already selected, rationale for selection.
5. Participants	
a. Inclusion criteria or desired participant characteristics	Discussion of scientific criteria vs. operational criteria, and strategy for reconciliation
b. Sample size	Or desired sample size
c. Recruitment strategy	**including role of operational partners**
6. Planned Interventions	
a. Intervention description	Who are the actors? What is the action being planned? Who is the target of the intervention? When is the intervention applied? What is the “dosage” of the intervention? What is the outcome the intervention intends to impact?
b. Development plan, if intervention not developed yet	If intervention has not yet been developed, describe the process that will be used to develop the intervention and the operational partner’s role in the process.
**c. Role of operational partner in intervention development and**/**or implementation**	
d. Fidelity assessment plan	How will the research team verify that the intervention is implemented as envisioned?
7. Measurements	Refer to reporting standards appropriate to specific study design, used as applicable (e.g. STROBE for observational studies; COREQ for qualitative research; CONSORT for RCT and similar designs; a repository of standards for other designs is available at www.equator-network.org)
8. Procedures	In addition to any details recommended by the reporting standards appropriate to specific study design used, **what role will the operational partners have in the development and execution of these procedures** **and in any other aspects of data collection**?
9. Data analysis	Refer to reporting standards appropriate to specific study design used (see #7 above)
10. *Potential challenges and limitations*	*Describe any anticipated challenges and the plan for addressing them*

This initial checklist reflects a combination of reporting elements that were either common across most reporting standards, or uniquely or especially relevant to partnered research (items in bold). A search for suitable reporting standards in the EQUATOR Network, PubMed, and SCOPUS databases yielded five sets of reporting standards (in addition to the CONSORT statement) containing elements that could be adopted or adapted to partnered research,^7^, ^8^, ^10^, ^12^–^14^ such as those drawn from participant action research, qualitative research, and adaptive trial designs. Elements listed in *italics* are optional—they provide important context, but do not adversely impact the potential scientific replicability of the study, nor are they likely to materially impact the outcome of the study if omitted. We acknowledge that, depending on the specific methodology used, not every element may apply to a given study.

Finally, reporting the role of the operational partners is of special importance in partnered research. As advocated by implementation science models,^6^ the context within which an intervention is implemented is of paramount importance to implementation success. Similarly, in partnered research, the context in which the research is occurring (the partner environment) is a known source of variance that must be accounted for as it would in any other type of research. Reporting the operational context in which the research is occurring (e.g., elements 3b–e, 4b, 6c) provides the level of transparency required to adequately assess the transportability of the research findings to other contexts. Thus, a formal protocol created in concert with the partners can help both the science and implementation of the project.

## CONCLUSIONS

Publishing protocols of partnered research can pose significant scientific challenges. Nevertheless, we believe that this endeavor is possible and has both scientific and operational value, especially as more research emerges, with the goals of accelerating the bench-to-bedside pipeline and implementing effective interventions in a timely manner. While operational partners may not have a clear understanding of the best methods to apply to any given study, they do understand the contextual and cultural idiosyncrasies unique to their sites. Clear, open, and structured communication can help achieve the rigor required in scientific work while providing actionable results to the sponsor that can be shared across multiple operational units. This communication is an active partnership that preserves the initial intent of the scientific work during the inevitable evolution of the study. The result of this partnership is research that optimally aligns rigorous investigation with operational needs.
